# Impact of the cervical cancer awareness months on public interest in Japan: A Google Trends analysis, 2012–2021

**DOI:** 10.1038/s41598-022-19798-x

**Published:** 2022-09-13

**Authors:** Hideharu Hagiya, Toshihiro Koyama, Fumio Otsuka

**Affiliations:** 1 Department of General Medicine, Graduate School of Medicine, Dentistry and Pharmaceutical Sciences, Okayama University, Okayama, 7008558 Japan; 2 Department of Health Data Science, Graduate School of Medicine, Dentistry, and Pharmaceutical Sciences, Okayama University, Okayama, 7008530 Japan

**Keywords:** Health care, Medical research, Oncology

## Abstract

The immunization and screening rates for human papillomavirus in Japan are lower than those in other countries. We aimed to evaluate the impact of cervical cancer awareness months on public attention using Google Trends analysis. Between 2012 and 2021, we analyzed the trends in relative search volumes (RSVs) for “Shikyuu-keigan” (cervical cancer in English) in Japan, during the cervical cancer awareness month (CCAM) in January and cervical cancer prevention awareness enhancement month (CCPAEM) in November. We performed a joinpoint regression analysis to identify a statistically significant trend change point. Additionally, we compared the mean RSVs of each awareness month with the rest of the year. Significant trend change points were observed, but none were found in CCAM and CCPAEM periods. Comparison of mean RSVs among CCAM, CCPAEM, and the rest of the months did not suggest any significant increases in RSVs during these awareness periods. In conclusion, CAM and CCPAEM did not raise public interest in cervical cancer in Japan. Although the results are based on internet users, the findings might suggest a need to develop a more effective and attractive approach to achieve the 90-70-90 targets of cervical cancer prevention by 2030.

## Introduction

Globally, cervical cancer is the fourth most common cancer among women^1^. It had an estimated > 600,000 new cases and > 300,000 deaths in 2020, most of which involved patients living in low- and middle-income countries^1^. More than 95% of cervical cancers are caused by human papillomavirus (HPV), which is transmitted mainly through sexual intercourse. The high efficacy and safety of HPV vaccines in preventing HPV infections and invasive malignancies have been proven by many observational studies, clinical trials, and post-marketing surveys^2–6^. To save lives from this preventable disease, the global strategy to eliminate cervical cancer as a public health problem was launched by the World Health Organization in 2020^7^. This policy involves primary prevention at ages of 9–14 years by HPV vaccination, secondary prevention at age > 30 years by cancer screening, and tertiary prevention as a treatment for invasive cancer at any age.

To eliminate cervical cancer, the 90-70-90 targets by 2030 are presently advocated^8^, in which it is recommended that 90% of girls should be fully vaccinated with HPV vaccine by 15 years of age, and 70% of women should be screened for cervical cancer by 35 years of age^9^. In Japan, the government initiated a public expense assistance system for HPV vaccination program targeting girls aged 12–16 years in 2010, for which bivalent and quadrivalent vaccines were officially approved in October 2009 and July 2011, respectively. Subsequently, HPV vaccination became one of the routine immunization program in April 2013, enabling females aged 12–16 years to receive the HPV vaccine for free of charge. In this situation, Japan’s HPV vaccination rate was approximately 70%^10^. However, because of reports regarding adverse reactions after HPV vaccination, the Japanese government publicized a postponement of its proactive recommendation^11^, and the HPV vaccination rate in Japan has dropped to less than 1%^12,13^. In addition, screening rates for cervical cancer in Japan have remained relatively low at approximately 40%^14,15^. At present, Japan is reportedly the only country in the literature that has experienced increasing trends of cervical cancer in both incidence and mortality in the last 10 years^16^.

Raising public awareness is highly important in facilitating HPV vaccination in Japan. An internet-based questionnaire survey performed in 2015 targeting Japanese females aged 16–20 years suggested that only 8.9% of unvaccinated girls knew the effectiveness of HPV vaccination in preventing cervical cancer^17^. To boost public awareness to alleviate the burdens of malignant diseases, cancer awareness months have been globally established, in which January is set as the cervical cancer awareness month (CCAM). Additionally, since 2010, November is the cervical cancer prevention awareness enhancement month (CCPAEM) in Japan, which has been endorsed by CancerNet Japan^18^. Recently, several studies have reported the usefulness of measuring public attention toward malignant diseases during the Cancer Awareness Months by the method of Google Trend analysis^19,20^. However, impacts of those awareness campaigns on public interests has not been fully evaluated in Japan. Therefore, this study aimed to evaluate the increase in public awareness of cervical cancer during CCAM and CCPAEM.

## Results

### Trends in the relative search volumes for the search term

**Figure 1:**
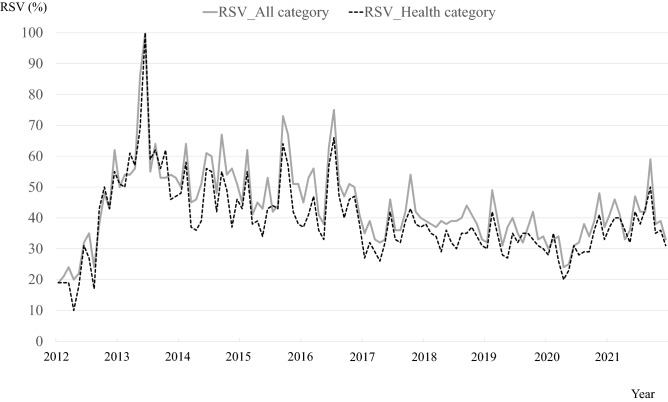
Monthly trends of relative search volume (RSV) for “Shikyuu-keigan” in Google Trends from 2012 to 2021. Data from the “All” and “Health” categories are depicted with solid and dotted lines, respectively.

The monthly trend of the relative search volume (RSV) for the search term “Shikyuu-keigan” from 2012 to 2021 is depicted in Fig. 1. Data from the “All” and “Health” categories showed almost identical fluctuations over time. The largest surge occurred in June 2013 (RSV at 100%), followed by September 2015 (RSVs at 73% in the “All” and 64% in the “Health” category), and July 2016 (RSV at 75% in the “All” and 66% in the “Health” category). Subsequently, there was no increase in the RSVs recorded at > 60%.

### Joinpoint analysis for the 10-year period

**Figure 2:**
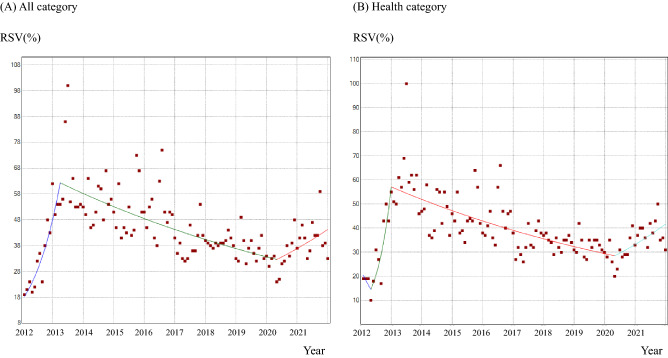
Monthly trends of RSV for “Shikyuu-keigan” in Google Trends by 2012 to 2021, by joinpoint analysis and search category. (**A**) In the “All” category, two joinpoints were observed in March 2013 and April 2020. (**B**) In the “Health” category, three joinpoints were observed in April 2012, December 2012, and April 2020. Details are given in Table 1.

The graphics and monthly percent changes (MPCs) in the RSV obtained by the joinpoint analysis are shown in Fig. 2 and Table 1, respectively. In the “All” category, there was an initial significant upward trend (MPC [95% confidence interval (CI)]; 9.1% [6.8 − 11.6]) with a peak in March 2013. Subsequently, the MPC gradually declined by April 2020 with MPC [95% CI] of − 0.8% [− 0.9 − − 0.6], which again showed an increasing trend until the end of the study period (MPC [95% CI]; 1.5% [0.2–2.8]). In the “Health” category, after a temporal decline, there was an initial increase up to December 2012 (MPC [95% CI]; 18.6% [10.7–27.0]), although the peak RSV was found in June 2013. The MPC thereafter decreased gradually as well and showed an increase again until the end. (MPC [95% CI]; 1.9% [0.5–3.4]).

**Table 1:** Monthly percentage changes (MPC) in relative search volumes (RSV) of “Shikyuu-keigan (cervical cancer)”, all through 2012 to 2021.

Category	Period 1		Period 2		Period 3		Period 4	
	Month/Year	MPC (%) [95% CI]	Month/Year	MPC (%) [95% CI]	Month/Year	MPC (%) [95% CI]	Month/Year	MPC (%) [95% CI]
ALL	Jan/2012–Mar/2013	9.1 [6.8 − 11.6]*	Mar/2013–Apr/2020	−0.8 [− 0.9−− 0.6]*	Apr/2020–Dec/2021	1.5 [0.2 − 2.8]*		
Health	Jan/2012–Apr/2012	−10.7 [−30.9–15.5]	Apr/2012–Dec/2012	18.6 [10.7–27.0]*	Dec/2012–Apr/2020	− 0.8 [− 0.9−− 0.6]*	Apr/2020–Dec/2021	1.9 [0.5–3.4]*

SE, standard error; CI, confidence interval.

*Significantly different from zero (*p* < 0.05).

### Joinpoint analysis for each year of 2012 to 2021

**Figure 3:**
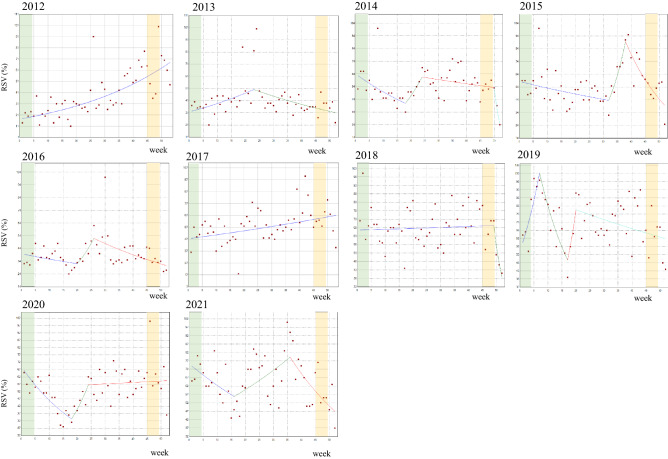
Weekly trend change analysis of relative search volumes in Google Trends by joinpoint regression in the “All” category by year from 2012 to 2021. The cervical cancer awareness month (CCAM) is depicted in green (week 1^st^ to 4^th^). The cervical cancer prevention awareness enhancement month (CCPAEM) is highlighted in yellow (week 45–48). Joinpoints are the time points at which statistically significant changes (*p* < 0.05) in the linear slopes were noted. Detailed data are presented in Table 2.

The graphics and weekly percent changes (WPCs) in RSV for the search time in the “All” category obtained by the joinpoint analysis are shown in Fig. 3 and Table 2, respectively. During the decade, we did not observe a clear surge in RSVs by means of the joinpoint analysis in either the CCAM or CCPAEM periods. During CCAM periods, there was only a temporal increase in RSVs in 2018, which did not form a cluster of increased RSVs. We found an aggregation of increased RSVs around the CCAM period in 2019, but its peak was at week 6 (middle of February) and the joinpoint analysis did not find a significant trend change point during the awareness month. During the CCPAEM periods, we detected temporal increases in RSVs in 2012 and 2020 without forming a monthly cluster of RSVs. In addition, we observed no apparent increase around the CCPAEM periods. We observed significant surges in RSVs around week 23 in 2013, week 38 in 2015, week 26 in 2016, and week 36 in 2021.

**Table 2:** Weekly percentage changes (WPC) in relative search volumes (RSV) of “Shikyuu-keigan (cervical cancer)” in “All category”, by each year, 2012–2021.

Year	Period 1		Period 2		Period 3		Period 4	
	Weeks	WPC (%) [95% CI]	Weeks	WPC (%) [95% CI]	Weeks	WPC (%) [95% CI]	Weeks	WPC (%) [95% CI]
2012	1–53	2.6 [2.0 − 3.2]*						
2013	1–23	2.0 [0.5 − 3.6]*	23–52	− 1.6 [− 2.6 − − 0.6]				
2014	1–18	− 2.5 [− 4.2 − − 0.8]*	18–24	7.1 [− 4.2–19.7]	24–50	− 0.6 [− 1.5–0.4]	50–52	− 31.7 [− 58.4–12.2]
2015	1–32	− 0.9 [− 1.7 − − 0.1]*	32–38	12.7 [− 0.9–28.2]	38–52	− 5.6 [− 8.1–− 3.0] *		
2016	1–20	− 1.1 [− 2.8–0.7]	20–26	8.4 [− 4.9–23.5]	26–52	− 2.0 [− 3.1 − − 0.9] *		
2017	1–53	0.7 [0.2–1.1]*						
2018	1–49	0.1 [− 0.2–0.4]	49–52	− 17.4 [− 33.9–3.3]				
2019	1–7	9.4 [0.6–18.9]*	7–17	− 7.1 [− 11.2–− 2.7]*	17–20	17.5 [− 28.5–93.1]*	20–52	− 0.8 [− 1.4–− 0.1]*
2020	1–18	− 4.0 [− 5.7–− 2.2]*	18–24	9.2 [− 2.7–22.6]	24–52	0.2 [− 0.7–1.0]		
2021	1–16	− 1.9 [− 4.0–0.3]	16–36	1.8 [0.2–3.4]*	36–52	− 3.2 [− 5.1–− 1.3]*		

SE, standard error; CI, confidence interval.

*Significantly different from zero (*p* < 0.05).

**Figure 4:**
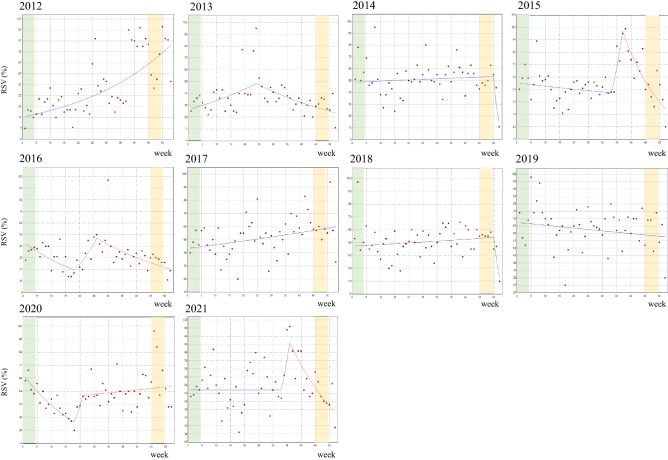
Weekly trend change analysis of relative search volumes in Google Trends by joinpoint regression in the “Health” category by year from 2012 to 2021. The cervical cancer awareness month (CCAM) is depicted in green (week 1–4). The cervical cancer prevention awareness enhancement month (CCPAEM) is highlighted in yellow (week 45–48). Joinpoints are the time points at which statistically significant changes (*p* < 0.05) in the linear slopes were noted. Detailed data are presented in Table 3.

The graphics and WPCs in RSV for the search time in the “Health” category obtained by the joinpoint analysis are provided in Fig. 4 and Table 3. Similar to the “All” category analyses, there was no uptrend of RSVs in these awareness months. During CCAM periods, there was also a one-point increase in RSVs in 2018, which did not form a significant RSV mass. During the CCPAEM period, we detected a cluster of increased RSVs in 2020. However, this was not determined to be significant by the joinpoint analysis. Outside these awareness months, we found significant surges of RSVs around week 24 in 2013, week 37 in 2015, week 26 in 2016, and week 36 in 2021. All these surges corresponded to those in the “All” category.

**Table 3:** Weekly percentage changes (WPC) in relative search volumes (RSV) of “Shikyuu-keigan (cervical cancer)” in “Health category”, by each year, 2012–2021.

Year	Period 1		Period 2		Period 3		Period 4	
	Weeks	WPC (%) [95% CI]	Weeks	WPC (%) [95% CI]	Weeks	WPC (%) [95% CI]	Weeks	WPC (%) [95% CI]
2012	1–53	3.0 [2.2 − 3.8]*						
2013	1–24	2.2 [0.8 − 3.8]*	24–52	− 2.2 [− 3.3 − − 1.1]*				
2014	1–50	0.2 [−0.3 − 0.7]	50–52	− 42.5 [− 70.5–12.1]				
2015	1–33	− 0.5 [− 1.3–0.4]	33–37	19.4 [− 12.7–63.3]	37–52	− 6.5 [−9.0–− 4.0]*		
2016	1–18	− 4.0 [− 6.5–− 1.3]*	18–26	10.4 [− 0.5–22.3]	26–52	− 2.8 [−4.2 − − 1.4]*		
2017	1–53	0.5 [0.1–1.0]*						
2018	1–50	0.3 [− 0.2–0.7]	50–52	− 32.2 [− 62.6–22.8]				
2019	1–52	−0.3 [− 0.7–0.1]*						
2020	1–18	−4.3 [− 6.2–− 2.4]*	18–21	18.7 [− 31.9–106.7]	21–52	0.4 [− 0.4–1.2]		
2021	1–33	0.0 [− 0.7–0.7]	33–36	13.8 [− 33.0–93.5]	36–52	− 4.0 [− 5.9–− 2.0]*		

SE, standard error; CI, confidence interval.

*Significantly different from zero (*p* < 0.05).

### Comparison of mean RSVs among CCAM, CCPAEM, and other weeks

The means and standard deviations for CCAM and CCPAEM periods in comparison with other weeks are shown in Table 4. In the “All” category, the Kruskal–Wallis test showed a *p-*value of 0.03; however, a significant difference was not observed among any mean RSVs after Bonferroni adjustment. Otherwise, there were no significant differences among the three categories. In the “Health” category in 2012, the mean RSV in CCAM was significantly lower than that in the other weeks (*p* = 0.028). In 2020, the mean RSV in CCPAEM was significantly higher than that in the rest of the week (*p* = 0.044).

**Table 4:** Comparison of mean relative search volumes (RSV) of “Shikyuu-keigan (cervical cancer)”, 2012–2021.

Category	Year	Mean RSV [standard deviation]			*P* values
		CCAM (week 1st to 4th)	CCPAEM (week 45th to 48th)	Other weeks	
All	2012	20.3 [3.5]	47.5 [12.9]	40.6 [21.1]	0.03*
	2013	37.0 [2.1]	37.5 [8.7]	42.0 [14.4]	0.62
	2014	58.8 [8.4]	50.3 [5.9]	54.1 [12.9]	0.47
	2015	53.8 [6.1]	52.5 [7.1]	56.3 [16.3]	0.98
	2016	34.0 [4.1]	39.5 [5.9]	40.0 [12.3]	0.31
	2017	48.3 [8.8]	60.0 [2.9]	59.0 [14.3]	0.16
	2018	75.5 [17.0]	68.0 [11.1]	66.7 [11.3]	0.67
	2019	68.5 [13.4]	66.5 [13.2]	74.1 [15.0]	0.40
	2020	58.0 [5.8]	71.3 [19.6]	52.0 [11.4]	0.07
	2021	71.5 [7.23]	63.3 [11.6]	65.0 [14.4]	0.50
Health	2012	17.8 [7.8]**	59.0 [17.4]	47.6 [27.4]**	0.012
	2013	38.0 [5.7]	37.8 [3.3]	42.3 [15.1]	0.69
	2014	64.0 [13.0]	51.8 [3.0]	56.1 [14.6]	0.21
	2015	60.5 [8.2]	47.0 [7.8]	57.7 [16.8]	0.22
	2016	38.0 [4.8]	33.5 [1.7]	35.3 [14.0]	0.61
	2017	53.8 [7.0]	62.3 [4.3]	59.0 [14.9]	0.41
	2018	64.0 [24.3]	58.3 [0.5]	52.5 [10.8]	0.61
	2019	65.0 [10.2]	61.5 [11.1]	66.4 [13.9]	0.65
	2020	59.8 [8.0]	75.0 [22.8]***	48.7 [10.8]***	0.007
	2021	59.8 [2.8]	62.3 [8.3]	64.8 [14.7]	0.72

*A significant difference was not observed among any mean RSVs after bonferroni adjustment.

**Mean RSV in CCAM was significantly lower than that in other weeks (*p* = 0.028).

***Mean RSV in CCPAEM was significantly higher than that in other weeks (*p* = 0.044).

*P* values were calculated by Kruskal–Wallis test. CCAM, Cervical Cancer Awareness Month (January); CCPAEM, cervical cancer prevention awareness enhancement month (November).

## Discussion

We demonstrated the public awareness of cervical cancer in Japan by employing Google Trends data for the last decade. In the long run, the highest RSV was observed in June 2013, followed by an increasing trend from April 2020 by the end of 2021. Although the data collected by the internet search does not necessarily reflect the entire awareness situation in the country, the year-by-year data suggest that public interests did not rise during these awareness months in Japan, implying that the awareness-raising activities in CCAM and CCPAEM were not powerful or influential enough to promote public attention in Japan. Japan is one of the advanced countries with high income, high literacy, and good accessibility to the internet, and an infodemiology study like our approach is relatively applicable. Thus, we consider the results of this study relatively accurately reflect the current state of public interest in cervical cancer in Japan; simply telling, there is a lack of awareness of cervical cancer prevention in Japanese society as a whole. However, beginning in April 2022, the public expense assistance system was resumed in Japan, and we assume that these awareness-raising campaigns will attract more attention than ever. 

Internet access to online searching engines, such as Google, has won the people's favor to gain knowledge on various topics. This greatly affected their attitudes, behaviors, intentions, and decisions at both the individual and national levels^21^. Even 10 years ago in 2012, more than half of the people in the United States answered that they had access to the internet for health-related issues^22^. Currently, data from the Google Trends platform have been broadly applied to quantify public awareness or interest in certain subjects^23–25^, indicating this approach to be a plausible indicator. Particularly in the cancer research field, a recent study evaluated the impact of World Cancer Day in Central and South American countries^26^. Another study investigated the influence of the coronavirus disease 2019 (COVID-19) pandemic on public interest in cancer screening^27^.

A review of social events that could have evoked increases in RSVs over the last decade should be discussed. In June 2013, when the highest RSV was achieved, the Japanese Ministry of Health, Labor, and Welfare decided to discontinue the proactive recommendation for HPV vaccination^28^, despite being already included in the lineup of the national immunization program. This government announcement was launched following the increased reports of vaccine-related adverse reactions and social concerns. In September 2015, when we observed a second momentary surge, the government joint meeting announced that it would continue to refrain from recommending HPV vaccination^29^. In July 2016, the Japanese government and two pharmaceutical companies were accused of the health hazards that occurred with HPV vaccination. According to the petition, 63 women aged 15 to 22 years filed a lawsuit with the courts in four cities in Japan. These instances could be largely associated with the rise in RSVs in each term.

Impact of the COVID-19 pandemic on public interest in cervical cancer is of interest to be considered. The World Health Organization declared the COVID-19 pandemic as the Public Health Emergency of International Concern at the end of January 2020. The present data indicated that MPC in RSV of cervical cancer in both All and Health categories showed significantly increasing trends since April 2020 (Fig. 2 and Table 1). Although the COVID-19 pandemic appears to have promoted public interest in cervical cancer, the causal relationship between these events is unexplainable. Generally considering, the unprecedented pandemic must have limited educational opportunities for preventive medicine for the public. In fact, an infodemiology study using the Google Trends reported a sharp drop in global online searching for cervical cancer after the declaration of the COVID-19 pandemic^30^.

To the best of our knowledge, this is the first attempt to investigate the impact of monthly awareness campaigns for cervical cancer on public awareness by utilizing Google trend data. The power of internet search data has become greater to estimate public interest and test a hypothesis in various fields. However, the limitations of the present study should be mentioned. First, Google Trends data is based on internet access, and thus, the present study included only those who had access to the internet and used Google to search^31^. However, the percentages of internet penetration between 2012 and 2019 were sufficiently high at 79.5% and 92.7%, respectively^32^. The market shares of Google searches in Japan remained over 70% during half of the study period^33^. This data suggests the robustness of Google Trends as a surrogate for public awareness. Second, the validity of searching and analysis methods used in this study should be evaluated. Although we consider the Japanese term “Shikyuu-keigan” to be the most familiar word for cervical cancer among the general population, whether it could comprehensively encompass the internet searching activity for the disease was uncertain. Also, collaborating Google trend data and joinpoint analysis to estimate public interest should be evaluated. However, in addition to our efforts ^34,35^, other researchers have applied this combination method in recent studies ^23–26^. Third, as indicated in a previous study^36^, Google Trends lacks full transparency and reproducibility. This is because RSVs are calculated based on non-published assumptions. Fourth, due to the nature of RSV data, non-scientific news, such as the death of the celebrity^37^, remarkably influences the results. Fifth, multiple comparisons may be associated with a statistical difference in the tested parameters. Despite these limitations, we believe our research could evaluate the public interest during the awareness months for cervical cancer in Japan, where vaccination and screening programs for preventable diseases need to be further promoted.

In conclusion, we highlighted that both global and domestic awareness months for cervical cancer failed to raise public interest during the last decade in Japan. Multifaceted and repeated approaches from another perspective are warranted to increase this trend, to ensure we meet the 90-70-90 targets for cervical cancer prevention by 2030.

## Methods

### Data source

Google Trends is a publicly available free data source based on Google search data. The open tool allows users to customize and obtain search term volumes that are entered into the Google search engine. It does not provide an absolute count of Google searches; rather, it provides an RSV with a scale of 0–100 (0 is the lowest and 100 is the highest popularity)^26,38–40^. This can indicate the degree of interest of a particular word at a certain time point. To date, the open database has beenwidely utilized to measure public attention in various fields, including healthcare research ^34,35,41–43^. This analysis allows us to estimate the relative popularity of a specific search terms of interest in each particular category (for example, “health,” “sports,” “business,” and so on), place (by country), and time range (various adjustments is available). In this study, we accessed the Google Trends platform and downloaded data in .csv format on February 1, 2022.

### Search input and variables

Our search protocol for Google Trends is provided in Supplementary Fig. 1, which was utilized in previous studies^26,34,35,40,44^. We searched in the Google Trends on 31st January 2022. The location of the search was selected for Japan, and we extracted data from 2012 and 2020, as well as each year of the study period. We put a Japanese word “Shikyuu-keigan” (cervical cancer in English) into the search term and chose the total category to extract the popularity of the term over a long period. In this way, we obtained monthly RSVs for the decade and weekly RSVs for each year and subjected them to statistical analysis to estimate whether the term was searched during the monthly awareness campaigns.

### Statistical analyses

To estimate the trend in the Google Trends RSV data, we applied a joinpoint regression model with the Joinpoint Regression Program (version 4.9.0.0, March 2021, Statistical Research and Applications Branch, National Cancer Institute, USA)^45^. The software identifies a joinpoint where a change in the temporal trend in the linear slope is statistically significant. We pre-set the criteria for finding joinpoints up to three points in this analysis: a minimum of 0 and a maximum of 3 joinpoints. MPCs and WPCs between trend-change points were determined with a 95% CI. To confirm the impact of the awareness months on public interest, we performed another analysis to estimate the difference in the mean weekly RSVs of the subjective and non-awareness months. As in a recent study^19^, the mean RSVs of January (CCAM, week 1st to 4th) and November (CCPAEM, week 45th to 48th) were compared to the mean RSVs for the rest of the year by the Kruskal–Wallis test. The threshold for statistical significance was defined as a *p*-value < 0.05, which indicated the level at which the slope differed from zero. The *p*-values for the Kruskal–Wallis test were adjusted using Bonferroni’s method.

### Ethical approval

This study protocol was approved by the Institutional Review Board of Okayama University Hospital with a waiver for informed consent, as the study intended to retrospectively analyze open, anonymized data (No. 1910-009). The authors assert that all procedures contributing to this work comply with the ethical standards of the relevant national and institutional committees on human experimentation and with the Helsinki Declaration of 1975, as revised in 2008.

## Supplementary Information

Supplementary Information.

## Data Availability

All data generated or analyzed during this study are included in this published article.
