# Supplementary material for: Impact of the cervical cancer awareness months on public interest in Japan: A Google Trends analysis, 2012–2021
Source: Sci Rep. 2022 Sep 13;12:15391. doi: 10.1038/s41598-022-19798-x (PMC9470073; doi:10.1038/s41598-022-19798-x)
Supplement: Supplementary file 1 — Supplementary Information. [file 41598_2022_19798_MOESM1_ESM.pptx]

## Slide 1
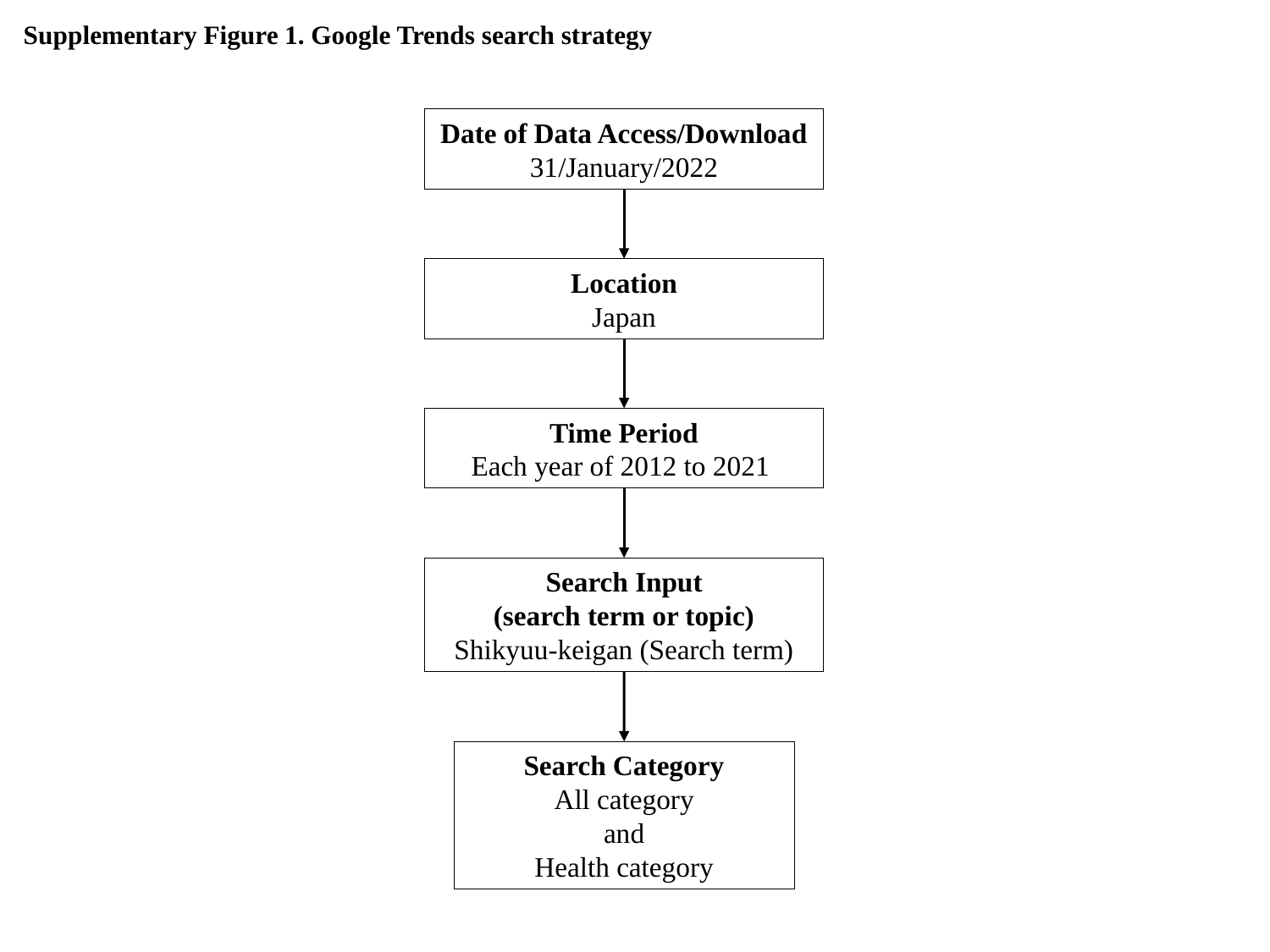

Supplementary Figure 1. Google Trends search strategy
Date of Data Access/Download
31/January/2022
Location
Japan
Time Period
Each year of 2012 to 2021
Search Input
(search term or topic)
Shikyuu-keigan (Search term)
Search Category
All category
and
Health category
